# Optimization of Quality Attributes and Atomic Force Microscopy Imaging of Reconstituted Nanodroplets in Baicalin Loaded Self-Nanoemulsifying Formulations

**DOI:** 10.3390/pharmaceutics10040275

**Published:** 2018-12-13

**Authors:** Géza Jakab, Viktor Fülöp, Tamás Bozó, Emese Balogh, Miklós Kellermayer, István Antal

**Affiliations:** 1 Department of Pharmaceutics, Semmelweis University; Hőgyes E. Street 7-9, Budapest 1092, Hungary; jakab.geza@pharma.semmelweis-univ.hu (G.J.); fulop.viktor@pharma.semmelweis-univ.hu (V.F.); balogh.emese@pharma.semmelweis-univ.hu (E.B.); 2 Department of Biophysics and Radiation Biology, Semmelweis University; Tűzoltó Street 37-47, Budapest 1094, Hungary; bozo.tamas@med.semmelweis-univ.hu (T.B.); kellermayer.miklos@med.semmelweis-univ.hu (M.K.)

**Keywords:** baicalin, self-nanoemulsifying system, atomic force microscopy, solubility improvement, graphical optimization, dynamic light scattering

## Abstract

The objective of the study was to develop baicalin loaded liquid self-nanoemulsifying drug delivery systems (BSNEDDS) and to characterize them by physicochemical methods in order to optimize the composition and quality attributes. Atomic force microscopy (AFM) was utilized to evaluate the morphological characteristics and size distribution of reconstituted nanoemulsion droplets with a new sample preparation method for the elucidation of individual nanodroplets without any signs of coalescence. Response surface methodology and desirability approach was used to select the optimized composition related to droplet size, zeta-potential, polydispersity index (PDI), and turbidity characteristics. Droplet size distribution measured by dynamic light scattering method was highly desirable with 52.87 ± 0.5322 nm, which was confirmed by AFM imaging. The optimized formula contains Peceol^®^ (14.29%, *w*/*w*), Kolliphor^®^ EL (57.14%, *w*/*w*), and Transcutol^®^ P (28.57%, *w*/*w*). Long-term stability analysis did not show any significant change in droplet size or PDI over the investigated period. More than 40.5-times solubility improvement was achieved with the optimized BSNEDDS correlated to solubility of baicalin in distilled water. In vitro dissolution studies at pH 1.2 and pH 6.8 were performed and revealed that the optimized BSNEDDS formula showed pH independent drug dissolution, and 100% of incorporated baicalin dissolved within five minutes in rapidly dispersing nanodroplets.

## 1. Introduction

*Scutellaria baicalensis Georgi* (*S. baicalensis G.*) is one of the fundamental herbs in traditional Chinese herbal medicine known as Huang Qin [1]. The root is officially listed in the Chinese Pharmacopoeia and was assumed in European Pharmacopoeia (Ph.Eur.) 9^th^ Edition (Ph. Eur. 9., 2018 [2]). The major components of the dried root are baicalin (d-glucuronic acid-5,6-dihydroxyflavone) and its aglycone, baicalein (5,6,7-trihydroxyflavone) [3]. In the scientific literature we can find numerous studies carried out showing that baicalin and its aglycone were effective medical agents with a variety of pharmacological activities such as anti-oxidant, anti-tumor, anti-ischemic, and anti-inflammatory effects [4].

Baicalin belongs to Class IV of the Biopharmaceutical Classification System (BCS) due to the extremely low water solubility (0.054 mg/mL) and lipophilicity (P_app_ = 0.037 × 10^−6^ cm/s, logP = 1.29) [5]. It is a weak acidic compound, with pKa values of 7.6 and 10.1 [6]. It can be seen from the chemical structure that both the glucuronide and flavone parts form intramolecular H-bonds, which can be responsible for the extremely poor water solubility and high melting point [7]. The stability of baicalin was evaluated in buffered aqueous solutions at different pHs (2.0, 3.0, 4.5, 6.8, 7.4, and 9.0) and temperatures (4, 25, and 40 °C). An acidic environment and low temperature were protective factors for the long term stability of this compound [8]. The absolute bioavailability of baicalin was 2.2 ± 0.2% after intravenous and oral administration to intact rats. It undergoes extensive intestinal glucuronidation and the fraction of baicalin and its conjugated metabolites excreted in urine appear to be negligible compared with the biliary excretion [9]. Different ATP-binding cassette (ABC) transporters were identified, which interfere in the enterohepatic recirculation of baicalin [10]. Various formulations were developed to counterbalance the low oral bioavailability of the drug. Solid dispersions, cyclodextrins, nanoemulsions, liposomes, and PEGylated lipid nanoparticles have already been investigated [11]. A baicalin-loaded nanohydrogel formulation showed promising performances in the topical treatment of skin wounds [12]. 

In recent years, Self-Emulsifying Drug Delivery Systems (SEDDS) have become an increasingly effective possibility for improving the oral bioavailability of poorly water-soluble drugs [13]. Self-emulsifying formulations are isotropic mixtures of natural or synthetic oils, lipophilic or hydrophilic surfactants, co-solvent(s) of the poorly water-soluble drug. These anhydrous preconcentrates spontaneously form oil-in-water emulsions upon mild agitation in the stomach [14]. Due to the numerous combination possibilities of different excipients, the Lipid Formulation Classification System (LFCS) was established by Pouton et al. in 2000 [15]. The LFCS classifies lipid-based formulations into four types according to their composition, droplet size, and characteristic features [15]. 

SEDDS can form colloidal micro/nanoemulsions (r < 100 nm) after dilution in the gastrointestinal tract (GIT) [16]. It is important to accurately specify the kind of colloidal dispersion used in a study, because this influences the physicochemical and functional properties. There are a lot of similarities between micro- and nanoemulsions (e.g., composition, fabrication, structure), but some differences can help distinguish between them [17]. The most significant difference between nanoemulsions and microemulsions is in terms of their thermodynamic stabilities. From a thermodynamic point of view, nanoemulsions are thermodynamically unstable, whereas microemulsions are thermodynamically stable systems [18]. There are numerous physicochemical phenomena which can lead to the breakdown of a nanoemulsion (e.g., creaming, flocculation, Ostwald ripening) [19]. By incorporating substances known as stabilizers, such as surfactants, texture modifiers, weighting agents, or ripening retarders, the long-term stability of nanoemulsions can be enhanced [20]. Although the physicochemical characterization of SEDDS and nanodroplets are well-known considering particle size distribution, zeta-potential, polydispersity index (PDI), turbidity, dilution robustness, and drug release, atomic force microscopy (AFM) had not yet been used for the morphological imaging of drug loaded nanodroplets [21,22]. We developed and optimized baicalin loaded self-nanoemulsifying drug delivery systems (BSNEDDS), which can overcome the limitations of conventional nanoemulsions, such as low patient compliance (poor palatability), unsuitability for delivery through hard or soft gelatine capsules (high water content), larger volume, and ambiguous long-term storage. SNEDDS provide easy manufacture and ability to scale, improved physical and chemical stability, and better patient compliance [23]. 

The aim of the study was to enhance the poor aqueous solubility and dissolution rate of baicalin via a liquid self-nanoemulsifying drug delivery system. Our scope was to find an optimized carrier system for baicalin, and to analyze and characterize the reconstituted nanoemulsion using AFM, droplet size, and Zeta-potential measurements, long-term stability tests, and in vitro dissolution studies. The development of a sample preparation method for AFM imaging of nanosized droplets was also in our interest.

## 2. Materials and Methods 

### 2.1. Materials

Baicalin (batch number: BA-16118) was supplied by Actin Chemicals, Inc. (Chengdu, China). The following materials were kindly donated by Gattefossé (Saint Priest, France) and were used as received: Capryol^®^ 90 (Propylene glycol monocaprylate), Labrafac^™^ Lipophile WL 1349 (Medium-chain triglycerides), Labrafil^®^ M 1944 CS (Oleoyl polyoxyl-6 glycerides), Labrasol^®^ (Caprylocaproyl polyoxyl-8 glycerides), Lauroglycol^™^ 90 (Propylene glycol monolaurate), Maisine^®^ CC (Glyceryl monolinoleate), Peceol^™^ (Glyceryl monooleate (type 40)), Transcutol^®^ P (Dyethylene glycol monoethyl ether). Kolliphor^®^ EL (Polyoxyl 35 hydrogenated castor oil), Kolliphor^®^ RH40 (Polyoxyl 40 hydrogenated castor oil), olive oil, and Emprove^®^ (Absolute ethanol) were purchased from Sigma Aldrich (St. Louis, MO, USA). Sunflower oil was supplied by Hungaropharma (Budapest, Hungary). Distilled water was prepared freshly whenever required. All other chemicals were of reagent grade. 

### 2.2. Solubility Studies

The thermodynamic solubility of baicalin was determined in distilled water, different oils, emulgents, and co-emulgents by saturation shake-flask method with minor modifications [24]. Firstly, an excess amount of baicalin was added to 2 g of each excipient in sealed vials. All samples were stirred (approx. 500 rpm) and thermostated (IKA RT-5 power heatable magnetic stirrer, IKA Work Inc., Wilmington, DE, USA) at 37 ± 1 °C for 24 h. Secondly a cycle of sedimentation (to achieve separation of the excess solid from the solution) was carried out for 24 h at 37 ± 1 °C. To improve the efficacy of phase-separation, the saturated supernatant of samples was centrifuged at 14,000 rpm for 15 min using a centrifuge (Herolab MicroGen 16, Herolab GmbH, Wiesloch, Germany) followed by removal of the incidentally undissolved baicalin from the supernatant by filtering it with a nylon membrane filter (0.22 μm, 25 mm, FilterBio^®^ NY, Labex Ltd., Budapest, Hungary). Samples were suitably diluted with absolute ethanol and drug concentration was obtained via UV spectroscopy at wavelength 279 nm using equivalent proportions of excipients as blank (Agilent 8453 UV-Visible Spectrophotometer, Agilent Technologies Ltd., Santa Clara, CA, USA). All experiments were repeated three times and results were calculated from the linear calibration of baicalin in absolute ethanol (R^2^ = 0.9991). Data are expressed as mean ± SD (µg/mL).

### 2.3. Screening of Surfactants for Emulsifying Ability

Various surfactants (Capryol^®^ 90, Kolliphor^®^ EL, Kolliphor^®^ RH 40, Labrafil^®^ M 1944 CS, Labrasol^®^, Lauroglycol^™^ 90) were screened for emulsification ability with the selected oily compound. According to pre-formulation experiments, different oil:emulgent *w*/*w* ratios (1:3, 1:4, 1:5, 1:6) were created, and selection of the emulgent was based on the results of droplet size and transmittance analysis. Briefly, 1 g of pre-concentrate was prepared in sufficient quantities, gently stirred, and heated to 37 ± 1 °C, promoting the homogenization process. The isotropic mixture, 0.1 mL, which was accurately weighed and diluted to 100 mL with distilled water, yielded fine emulsions. Emulsions were equilibrated for 2 h at room temperature before measuring their transmittance by Agilent 8453 UV-Visible Spectrophotometer at wavelength 633 nm using distilled water as blank. The droplet size (Z-avg) distributions and polydispersity indices were measured by DLS (dynamic light scattering) method with the instrument Zetasizer Nano ZS^™^ (Malvern Instruments Ltd., Malvern, UK). Measurement settings were: automatic mode, NIBS (none-invasive-back-scattering) 173°, 30 sub runs/measurements, run duration of 10 s, automatic laser position selected, 4.65 mm position from the bottom of the cuvette, attenuation setting of attenuator 9 was selected automatically. Five measurements with 30 runs were performed for every sample and the mean ± SD values are reported in this article for all DLS parameters, including intensity-weighted mean hydrodynamic diameters (Z-avg) and polydispersity indices (PDI). 

### 2.4. Construction of Ternary Phase Diagram

In order to identify the self-nanoemulsifying compositions with the desired droplet size (Z-avg < 200 nm), a ternary phase diagram was constructed [25]. For every mixture, the surfactant and co-surfactant (S_mix_) ratios were varied from 1:1, 1:2, 1:3, and 2:1. 2 g of the oil and S_mix_ in different *w*/*w* ratios (1:9, 1:8, 1:7, 1:6, 1:5, 1:4, 1:3, 1:2, 1:1, and 2:1) were measured, blended for 1 h at approximately 500 rpm (IKA RT-5 power heatable magnetic stirrer), and heated at 37 ± 1 °C. Compositions were evaluated for nanoemulsion formation by dropping 100 μL of each of the 40 mixtures in glass beakers containing 100 mL distilled water that was maintained at 37 ± 1 °C, followed by Z-avg measurements (for setting parameters see Section 2.3). All experiments were repeated three times and the ternary phase diagram was constructed using the ProSim ternary diagram drawing application (ProSim, Toulouse, France).

### 2.5. Preparation of Self-Nanoemulsifying Formulations without (SNEDDS) and with Baicalin (BSNEDDS)

In all cases, oil, emulgent, and co-emulgent were thermostated at 37 ± 1 °C and stirred (approximately 500 rpm) in different *w*/*w* ratios by a heatable magnetic stirrer. After a 1-h homogenization cycle, they were stored at room temperature in sealed vials until further use. BSNEDDS were prepared by the above-mentioned method, but at the end of the homogenization cycle, 5 mg of baicalin was added to 2 mL of the pre-concentrate (C = 2.5 mg/mL). 

### 2.6. Optimization of SNEDDS Pre-concentrates

In order to reduce the number of trials needed in the optimization of SNEDDS formulation, and to characterize the relationship between the formulation factors and the output variables, a response surface methodology based on Face Centered Central Composite Design was utilized. The amount of oil and S_mix_ ratio can significantly influence the quality and performance of a nanoemulsion, so different oil:S_mix_ (1:8, 1:6, 1:4) ratios and emulgent:co-emulgent (1:1, 2:1, 3:1) ratios were analyzed as formulation variables (X_1_, X_2_). In the study, a three-level (coded as +1, 0, −1) factorial design for the optimization of two variables with 13 runs (5 center points) was applied. Droplet size (Y1), transmittance (Y2), zeta-potential (Y3), and PDI (Y4) were selected as responses. Experiments were run in random order to increase the predictability of the model. The modelling of corresponding response surfaces was carried out using second order models, which can describe the surface curvature with the following polynomial equation:

(1) $$Y=b_{0}+b_{1}x_{1}+b_{2}x_{2}+b_{12}x_{1}x_{2}+b_{11}x_{1}^{2}+b_{22}x_{2}^{2}$$

where *Y* is the dependent variable; $b_{0}$ the intercept is the arithmetic average of all quantity outcomes of 13 runs; *x* are the independent variables ($x_{1}$ oil:S_mix_ ratio, $x_{2}\;$ emulgent:co-emulgent ratio) and *b* parameters mark the regression coefficients characterizing the main (*b*_1_, *b*_2_), the quadratic (*b*_11_, *b*_22_), and the interaction effects (*b*_12_).

A substantial goal of an optimization process is to find the most desirable set of conditions. Optimization of multiple responses was carried out by graphical optimization, which set minimum or maximum limits for each response then created an overlay graph highlighting an area of desired operability. The optimization and statistical experiments were designed and evaluated using the Design-Expert^®^ software, version 7.0.0 (Stat-Ease^®^ Inc., Minneapolis, MN, USA, 2005).

### 2.7. Characterization of Optimized BSNEDDS

#### 2.7.1. Droplet size, Transmittance, PDI, and Zeta-Potential Measurements

Droplet size, transmittance, and PDI were determined according to Section 2.3. Zeta-potential was measured using the Zetasizer Nano ZS^™^ (Malvern Instruments Ltd., Malvern, UK) by diluting 0.1 mL of optimized BSNEDDS with 100 mL of distilled water. The evaluation was based on Laser Doppler Micro-electrophoresis using the Smoluchowski model. The selection of the attenuator level and the position of the optics was set automatically. The instrument was operated by automatic selection of voltage based on the measured conductivity of the sample. The analysis was carried out at 37.0 °C in clear, disposable folded-capillary zeta cells. Five measurements with minimum 10 runs/sample were performed for each sample, the mean values ± SD (mV) are reported.

#### 2.7.2. Determination of the Thermodynamic Solubility of Baicalin in Optimized SNEDDS

2 g of selected oil, emulgent, and co-emulgent were thermostated and homogenized (37 °C, approximately 500 rpm, 1 h) in optimized ratios by a heatable magnetic stirrer. An excess amount of pure baicalin was added to the best pre-concentrate in sealed vials. Henceforth, the investigation was fulfilled as detailed in Section 2.2.

#### 2.7.3. Cloudpoint Measurement

Cloudpoint is the temperature above which an aqueous solution of a water-soluble surfactant becomes turbid. The optimized formula was diluted with distilled water to 100 times and placed in a water bath where the temperature was increased gradually (1.0 °C/min). Cloudpoint was recorded as the temperature at which the diluted formulation turned cloudy (visual perception). 

#### 2.7.4. Effect of Dilution on Droplet Size and PDI

The optimized formulation was evaluated for robustness of dilution. The pre-concentrate was diluted to 50, 100, 500, and 1000 times by distilled water in 100 mL Erlenmeyer-beakers with continuous stirring at 37 ± 1 °C. Distribution parameters were measured using the dynamic light scattering method as described above (Section 2.3).

#### 2.7.5. Long-Term Physical Stability of Nanoemulsions

0.2 mL of optimized BSNEDDS was dropped by automatic pipette into 100 mL distilled water at 37 ± 1 °C, with continuous stirring for 1 h at approximately 500 rpm (IKA RT-5 power heatable magnetic stirrer). After several minutes of mild stirring, an intrinsic droplet size and PDI were measured by Zetasizer Nano ZS^™^ (for setting parameters see Section 2.3). The Erlenmeyer flask with stopper was stored at room temperature and protected from direct sunlight until further investigation. Determinations were performed at 37.0 °C throughout the storage time (1 day, 3 days, 7 days, 14 days, 21 days, 28 days). 

#### 2.7.6. Atomic Force Microscopy (AFM)

##### Sample Preparation for AFM Imaging

The BSNEDDS pre-concentrate was diluted 10^7^-fold with distilled water. Five µL of this emulsion was dropped on a freshly cleaved mica surface and was frozen by pouring approximately 30 mL liquid N_2_ onto it. The sample was immediately placed in the lyophilizator chamber (CoolSafe^™^ 110-04 freeze dryer, ScanVac, Lillerød, Denmark), pre-cooled to −60 °C, and lyophilized at the following parameters: 10 min freezing at −40 °C, then drying at 0.020 hPa vacuum chamber pressure for 18 h. The shelf temperature was set at 15 °C for 1 h, 20 °C for 1 h, 30 °C for 8 h, and 40 °C for 4 h. Freeze-dried samples were stored protected from light at ambient humidity at 25 ± 1 °C and examined within 4 h of the end of the lyophilization process.

##### AFM Imaging and Analysis

Lyophilized samples were imaged in non-contact mode with a Cypher S instrument (Asylum Research, Santa Barbara, CA, USA) at 1–2 Hz line-scanning rate in air using a silicon cantilever (OMCL AC-160TS, Olympus, Tokyo, Japan) and oscillated at its resonance frequency (typically 300–320 kHz). Temperature during the measurements was 29 ± 1 °C. Images were analyzed by using the built-in algorithms of the AFM driving software (IgorPro, WaveMetrics Inc., Lake Oswego, OR, USA). AFM amplitude-contrast images are shown in this paper. To determine height variations, height-contrast data were used.

### 2.8. In Vitro Dissolution Study and Nanoemulsifying Ability Test of BSNEDDS

Dissolution testing was performed using the Hanson SR-8 Plus^™^ Dissolution Test Station (Hanson Research, Los Angeles, CA, USA) by the paddle (United States Pharmacopeia apparatus 2) method at 50 rpm and 37 ± 0.2 °C. The formulations were screened in 500 mL of aqueous-based dissolution media at pH 1.2 (Ph.Eur.9.) and pH 6.8 (Ph.Eur.9.). Pure baicalin and liquid BSNEDDS (equivalent to 5 mg of baicalin) were exposed to the media, and at predetermined time-points, 5 mL of samples were withdrawn and filtered from the media through 5 µm pore size membrane full-flow filters by Hanson AutoPlus Multifill collector (Hanson Research). After every sampling, media replacement was accomplished with 5 mL of fresh buffer solution. Dissolution studies were completed in triplicate and the dissolved drug content (%) ± SD was analyzed by UV-Vis spectrophotometry at 279 nm against equivalent proportions of excipients as blank. Withdrawn and filtered samples were characterized for droplet size and PDI as described for liquid BSNEDDS in Section 2.3. 24 h of visual observation was fulfilled for any precipitation or phase separation. The hydrophobic API favours being dissolved in the droplet of nanoemulsion, and this state (preceded by filtration and dispergation) was measured by UV-Vis spectroscopy.

## 3. Results 

### 3.1. Solubility Studies

Oil represents one of the most important excipients in SNEDDS formulations. To achieve optimal drug loading, it is essential to choose the oil having the greatest solubilizing capacity [26]. Amongst the various oils that were examined, Peceol^®^ showed the highest solubilizing capacity (719.1 ± 83.05 µg/ml) for baicalin, followed by Labrafac^™^ Lipophile WL 1349 and Maisine^®^ CC. The investigated natural oils were unsuitable for dissolving the target amount of baicalin. This observation is in accordance with the existing literature [27]. Based on solubility studies, Peceol^®^ was the ideal choice as an oily phase. 

**Figure 1:**
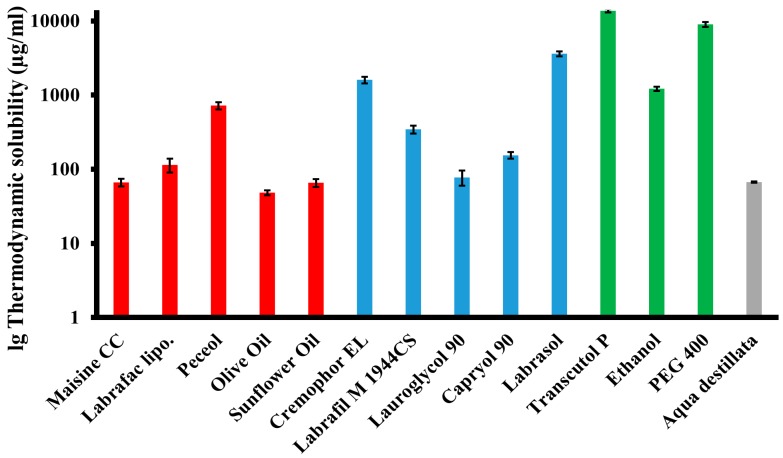
Thermodynamic solubility of baicalin in various oils (red), emulgents (blue), co-emulgents (green), and distilled water (gray). (Mean ± SD; n = 3).

Selection of a surfactant depends on several factors, such as solubilizing capacity for the API, the efficiency and rapidity of emulsification of the oil, and safety [28]. Non-ionic surfactants are considered less toxic than ionic ones. Due to these considerations, we analyzed five non-ionic surfactants with various hydrophilic-lipophilic balance (HLB) values. The highest solubility of baicalin was obtained in Labrasol^®^ (3609.4 ± 277.39 µg/mL) followed by Kolliphor^®^ EL and Labrafil^®^ M 1944 CS. Solubility studies are necessary, but conditions were not sufficient for selecting an emulgent, so Kolliphor^®^ EL, Labrafil^®^ M 1944 CS, and Labrasol^®^ were tested further for emulsification ability (see Section 3.2) [29]. The role of the co-surfactant together with the surfactant is to lower the interfacial tension, and to facilitate dissolving large amounts of API in the oily phase [26]. Drug absorption and dispersibility can also be improved by prudent selection of co-emulgent [30]. Transcutol^®^ P, an ether derivate, revealed extremely high (13742.5 ± 691.74 µg/mL) solubilizing capacity, and was therefore chosen as a co-surfactant for further investigations. The results of the solubility study are presented in Figure 1. 

### 3.2. Screening of Surfactants for Emulsifying Ability

**Figure 2:**
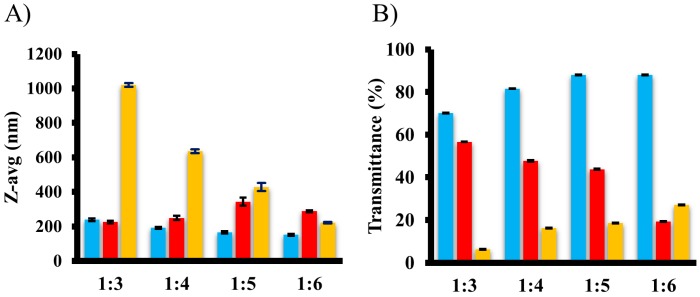
Emulsification ability of Kolliphor^®^ EL (blue), Labrafil^®^ M 1944 CS (red), and Labrasol^®^ (yellow) with Peceol^®^ at different *w*/*w* ratios characterized by droplet size (**A**) and transmittance (**B**) analysis. (Mean ± SD; n = 3).

Three emulgents with various molecular structures and HLB-values were chosen in order to screen their ability to emulsify the oily phase by droplet size (Figure 2A) and turbidity analysis (Figure 2B). The droplet size of emulsions of Labrasol^®^ and Kolliphor^®^ EL demonstrated reduction with increased emulgent content, while Labrafil^®^ M 1944 CS did not indicate any significant droplet size shift when mixed with various oil ratios. All Labrasol^®^-containing formulations demonstrated unacceptable self-emulsification efficiency and turbid systems were generated. Kolliphor^®^ EL showed the best emulsification ability and produced the most desired droplets with 151 ± 1.2871 nm at Peceol^®^ Kolliphor^®^ EL 1:6 *w*/*w* ratio. Turbidity analysis revealed a clear augmentation of transmittance with increased oil:emulgent ratios for Kolliphor^®^ EL and Labrasol^®^, however Labrafil^®^ M 1944 CS expressed a sharp decline. It has been reported that the required HLB value to form o/w nanoemulsion is greater than 10 [31]. The very poor emulsifying ability of Labrafil^®^ M 1944 CS can be attributed to its low HLB-value (HLB = 3). Labrasol^®^ (HLB = 14) exhibited better affinity for the oil phase compared to Labrafil^®^ M 1944 CS, although the best compatibility was shown with Kolliphor^®^ EL (HLB = 12). This indicates that nanoemulsification was also influenced by other factors besides HLB value, such as the chemical structure, saturation, and chain length of the surfactant [32]. Furthermore, it was demonstrated that Kolliphor^®^ EL has an inhibitory impact on different CYP-enzymes and on intestinal P-glycoprotein, which are desirable to enhance the bioavailability [33]. We have selected Kolliphor^®^ EL as giving the greatest transmittance and lowest droplet size with increased oil:emulgent ratios. In consideration of the aforementioned results and observations, we can conclude that Peceol^®^, Kolliphor^®^ EL, and Transcutol^®^ P is the best choice for designing baicalin containing SNEDDS.

### 3.3. Construction of Ternary Phase Diagram

**Figure 3:**
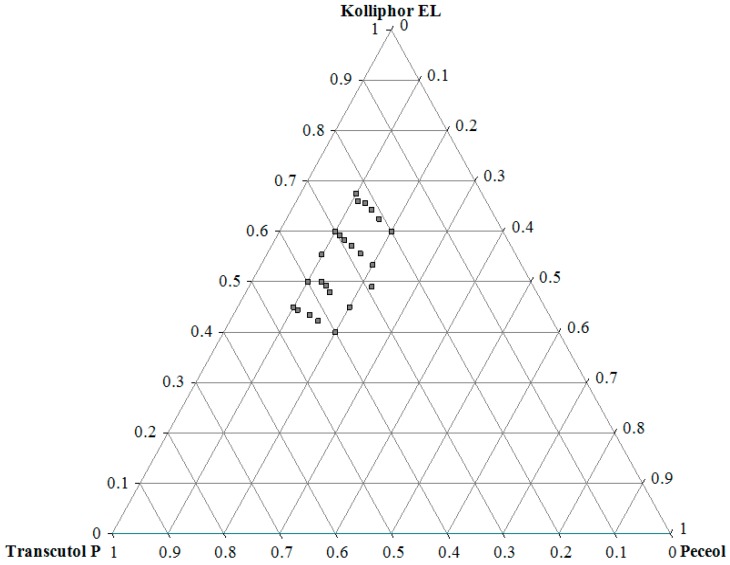
Ternary phase diagram of Peceol^®^, Kolliphor^®^ EL, and Transcutol^®^ P based on droplet size and transmittance analysis. The area bordered by grey squares represents the self-nanoemulsifying region. (n = 3).

The ternary phase diagram of different formulations of SNEDDS, which was prepared by varying the concentration of Peceol^®^, Kolliphor^®^ EL, and Transcutol^®^ P, is demonstrated in Figure 3. The ternary phase diagram was constructed based on solubility and emulsification studies to identify the nanoemulsifying region (Z-avg < 200 nm, T% > 90%), and the composition also helps to determine the concentration range of components for the formation of a nanoemulsion. Compositions containing more than 22% oil phase were found to be out of the nanoemulsifying region. It was observed that at least 40% surfactant is required for producing droplets under 200 nm. 

### 3.4. Optimization of SNEDDS Pre-concentrates

Independent variables and their levels and dependent variables with goals used in Face-centered experimental design are indicated in Table 1. Thirteen experiments were designed to understand the influence of formulation variables (oil:S_mix_ and emulgent:co-emulgent ratios) affecting droplet size, transmittance, zeta-potential, and PDI (Table 1). The values of responses Y_1_ (Z-avg (nm)), Y_2_ (Transmittance (%)), Y_3_ (Zeta-potential (mV)), and Y_4_ (PDI) ranged from 20.3 to 200.8 nm, 78.7 to 100%, −33.8 to −15.9, and 0.25 to 0.69, respectively. The ratio of maximum to minimum for responses Y_1_, Y_2_, Y_3_, and Y_4_ was found to be 9.892, 1.271, 0.47, and 2.76, respectively. Power transformation was not required for these responses and no aliases were found for the Quadratic model, and in all cases the Sequential Model Sum of Squares detected the Quadratic model as being significant. The models were validated by one-way analysis of variance (ANOVA), lack of fit, and R^2^ tests. The Model F values for responses Y_1_, Y_2_, Y_3_, and Y_4_ were 120.36, 66.24, 50.03, and 47.72, respectively, which implied that each model was significant. The final polynomial equations and a summary of the regression analysis on all the responses are presented in Table 2. The main effects (*b*_1_, *b*_2_) represent the average result of changing one variable at a time from its low level to its high level while the other is kept fixed. The interaction terms $\left(b_{12}\right)$ show how Y_1_–Y_4_ change when two variables are simultaneously changed, while the quadratic terms (*b*_11_, *b*_22_) symbolize nonlinearity. The positive sign of the coefficients indicates a synergistic effect on responses, while the negative sign expresses an antagonistic effect. Our analysis also revealed non-significant lack of fit test results (*p* > 0.05) for all the measured responses, which strengthened the reliability of the models. 

Analysis of standardized main effects (only significant values *p* < 0.05 are discussed) showed that droplet size (Y_1_) was affected by the synergistic effect of oil:S_mix_ ratio, antagonistically influenced by emulgent:co-emulgent ratio, the interaction term between oil:S_mix_ and emulgent:co-emulgent ratio, and quadratic terms of oil:S_mix_ ratio. Transmittance (Y_2_) was synergistically affected by emulgent:co-emulgent ratio and interaction between oil:S_mix_ and emulgent:co-emulgent ratio, and an antagonistic effect was found with oil:S_mix_ ratio. In the case of Zeta potential (Y_3_), we found a positive correlation with oil:S_mix_ ratio and its quadratic term, and a negative correlation with emulgent:co-emulgent ratio. PDI (Y_4_) showed a significant synergistic effect with oil:S_mix_ ratio and emulgent:co-emulgent ratio. Based on these results, it is obvious that all the factors contribute in determining the characteristics of SNEDDS. 

**Figure 4:**
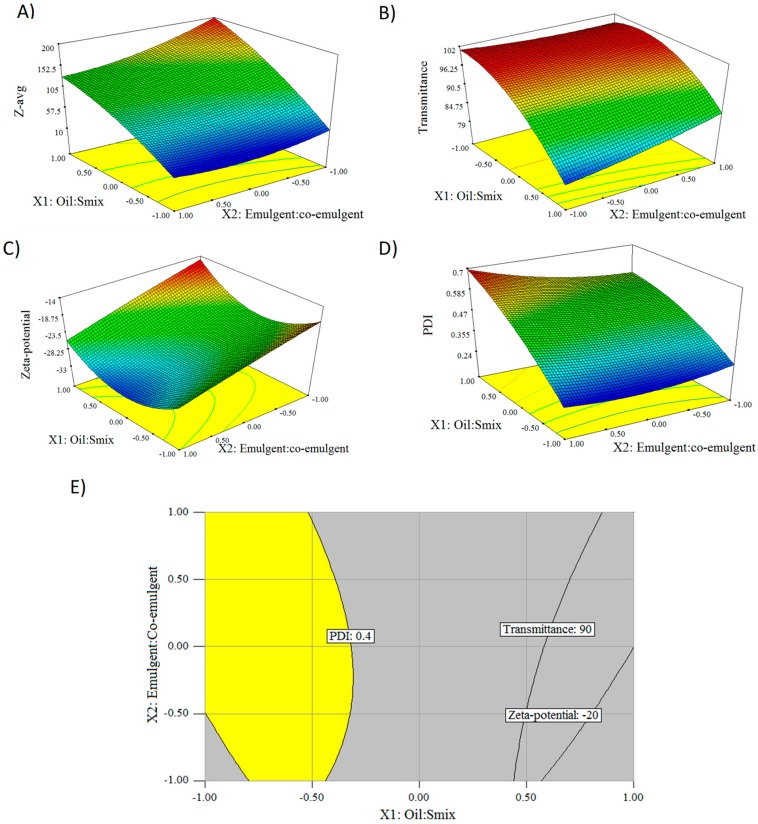
3D response surface plots for effect of oil:S_mix_ ratio (X_1_) and emulgent:co-emulgent ratio (X_2_) on Z-avg (**A**), Transmittance (**B**), Zeta-potential (**C**), and PDI (**D**). Overlay plot (**E**) for various oil:S_mix_ and emulgent:co-emulgent ratios, where the area that satisfies the constraints is yellow, while the area that does not meet criteria is grey.

Response surface method (RSM) designs help to quantify the relationships between one or more measured responses and the vital input factors. The generated 3D plots show how any of two factors affects the response. Figure 4A displays the effect of oil:S_mix_ (X_1_), emulgent:co-emulgent (X_2_), and their interaction on droplet size (Y_1_). When both oil:S_mix_ (1:8) and emulgent:co-emulgent were low (1:1), a mean droplet size of 27.7 ± 0.12 nm was observed. By increasing oil content while keeping X_2_ at 1:1, droplet size was increased to 200.8 ± 1.64 nm. As the amount of surfactant and co-surfactant mixture increased, so decreased the droplet size. It can be attributed to the fact that strong localization of surface-active agents at the oil–water interface reduces the interfacial free energy. The higher the oil content, the more extensive the total interfacial area to be stabilized, and the amount of surfactant molecules are not sufficient to cover the oil droplets and lower interfacial tension at the oil–water interface. The lowest droplet size (20.3 ± 0.08) was achieved at a low oil:S_mix_ level (1:8) and at high emulgent:co-emulgent ratio (3:1). The 3D response surface plot of transmittance is demonstrated in Figure 4B. Formulations are considered transparent if the percentage transmittance is above 90%, which is due to the fact that droplet size is not larger than 25% of the wavelength of incident light [34]. Low level (−1) of oil:S_mix_ ratios were favourable for producing perfectly transparent nanoemulsions (100% and 99.9%), irrespective of the emulgent:co-emulgent ratio. As the amount of oil decreased in the formulation, the transmittance increased, and vice versa. Examining the responses, the minimum transmittance value (78.7 ± 0.08%) was detected at a high level of oil:S_mix_ and at a low level of emulgent:co-emulgent. Figure 4C illustrates the relationship between oil:S_mix_ and emulgent:co-emulgent ratios on Zeta-potential. The lowest Zeta-potential (−33.8 ± 0.83 mV) was observed at the middle level of oil:S_mix_ and high level of emulgent:co-emulgent ratio. This electrokinetic potential was decreased by decreasing the ratio of co-surfactant. This phenomenon might be explained by the insufficient co-surfactant concentration, because the co-surfactant plays a special role in reduction of interfacial tension and providing flexibility of the interfacial film [26]. The highest Zeta-potential (−15.9 ± 0.72) was detected at high oil:S_mix_ and low emulgent:co-emulgent level. The negative surface charge of dispersed nanoemulsions can be explained by the chemical structure of Peceol. Peceol composed of glyceryl monooleate contains free fatty acids, due to manufacturing or hydrolysis of glyceryl monooleate during storage. According to the technical data sheet from the manufacturer, free glycerol content in Peceol is ≤ 6% and acid value is 3.0 mg KOH/g, which meets the requirements of Eur. Ph. and United States Pharmacopoeia (USP). Fatty acids in solutions can deprotonate to carboxylate anions [35]. The analysis of PDI (Figure 4D) pointed out parallel relationships with the droplet size measurements and some differences as well. The emulgent:co-emulgent ratio slightly influenced the polydispersity indices, while the effect of various oil:S_mix_ ratios were significant (p < 0.001) on PDI. The narrowest droplet size distribution (0.25) was found when oil:S_mix_ was at low level and emulgent:co-emulgent was high. We can conclude that higher oil content of the pre-concentrate produces a nanoemulsion with wider droplet size distribution. The desired PDI value should be less than 0.400, which implies a uniform particle size distribution [36]. 

All the measured responses were graphically optimized using the optimization module of the software. With multiple responses it is necessary to find regions where requirements simultaneously meet the critical properties. By superimposing or overlaying critical response contours on a contour plot, it is possible to visually search for the best compromise. The minimum and maximum acceptable responses were determined according to the scientific literature: droplet size < 200 nm, transmittance >90%, Zeta-potential < ±20 mV, PDI < 0.40. The overlay plot can be seen in Figure 4E, where the area that satisfies the constraints is yellow, while the area that does not meet criteria is grey. We selected our optimized formulation from the middle of the yellow labelled area of the overlay plot. The optimized SNEDDS contained Peceol^®^ (14.29%, *w*/*w*), Kolliphor^®^ EL (57.14%, *w*/*w*), and Transcutol^®^ P (28.57%, *w*/*w*). The predicted values for responses Y_1_, Y_2_, Y_3_, and Y_4_ were 59.63 nm, 99.51%, −26.50 mV, and 0.36, respectively. Three batches of the optimized formulation were prepared and measured, 52.87 ± 0.5322 nm, 99.87 ± 0.18%, -25.42 ± 0.710 mV, and 0.335 ± 0.004. The predicted and observed values of responses were in very close correlation, indicating the reproducibility of the model. 

### 3.5. Characterization of Optimized BSNEDDS

#### 3.5.1. Droplet Size, Transmittance, PDI, and Zeta-Potential Measurements. Comparison of Loaded and Unloaded SNEDDS

The droplet size, transmittance, PDI, and Zeta-potential of optimized BSNEDDS was measured. It is a well-known fact that droplet size is an essential feature and can significantly influence the in vivo fate of a nanoemulsion. The globule size of an emulsion also determines the rate and extent of drug release [37]. The Z-avg was highly desirable with 86.75 ± 0.3553 nm, likewise the PDI value with 0.403 ± 0.007, indicating that the system had narrow droplet size distribution and indicating improved drug dissolution kinetics. Transmittance of the diluted sample was 99.93 ± 0.13%. In general, when the Zeta-potential of an emulsion is high (> ±20 mV), the repulsive forces exceed the attractive forces, resulting in a relatively stable system. The long-term stability of a nanoemulsified system can be estimated by examining the fluctuation of the Zeta-potential [38]. The Zeta-potential of optimized BSNEDDS was −24.3 ± 1.44 mV and the specific conductivity was found to be 0.0135 ± 0.002 mS/cm.

We confirm that some features of unloaded SNEDDS have changed due to drug loading, but the experiments supported the suitability of loaded SNEDDS to deliver baicalin. PDI and droplet size were compared at two pH values in case of both unloaded and loaded SNEDDS (Table 3) to demonstrate the effect of pH and highlight if there is any difference between the formulas due to drug loading. From the results it is clear that pH has no effect on droplet size and PDI, but drug loading has a consequence. The colloid dispersion formed from loaded SNEDDS undoubtedly meets the quality criteria and characteristics for a nanoemulsion (droplet size < 200 nm, PDI < 0.40). It was pointed out that the drug itself didn’t influence the self-dispersing process, as dilution with a dispersing media self-emulsification takes place in seconds.

#### 3.5.2. Determination of the Thermodynamic Solubility of Baicalin in Optimized SNEDDS

The thermodynamic solubility of baicalin in optimized SNEDDS was 2714.3 ± 113.8 µg/mL. It indicated 40.5-times solubility improvement correlated with solubility in distilled water (67.0 ± 1.6 µg/mL). The hydrophobic API favoured being dissolved in the droplet of nanoemulsion, and this state (preceded by filtration and dispergation) was measured by UV-Vis spectroscopy.

#### 3.5.3. Cloudpoint Measurement

A special property of non-ionic surfactants is cloudpoint, the temperature above which the surfactant phase separates and precipitates out of solution. The cloudpoint should be higher than 37 °C in the ideal case because of the risk of irreversible phase separation in the body [39]. The cloudpoint for BSNEDDS was 71–73 °C, providing stabile nanoemulsion in vivo at physiological temperatures. 

#### 3.5.4. Effect of Dilution on Droplet Size and PDI

After oral administration of SNEDDS, nanoemulsion formation upon dilution takes place in vivo. It is a relevant aspect to demonstrate that uniform nanoemulsions are formed in terms of various dilutions, and these nanoemulsified systems do not show any sign of phase separation or precipitation. The drug may precipitate in vivo at higher dilutions, which might significantly affect drug absorption [40]. All emulsions were found to be in the acceptable nanoemulsion region (Z-avg. < 200 nm, T > 90%, PDI < 0.4), proving their robustness to dilution. After 24 h of inspection, neither phase separation, nor precipitation, was observed. In vitro results showed that BSNEDDS in different dilutions form uniform nanoemulsions and drug solubility in BSNEDDS was adequate (no precipitation).

#### 3.5.5. Long-Term Physical Stability of Nanoemulsions

The long-term physical stability of BSNEDDS was evaluated by droplet size, PDI measurements, and macroscopic emulsion observation for a month. There are a lot of various physicochemical phenomena which can lead to the breakdown of a nanoemulsion (e.g., creaming, flocculation, Ostwald ripening), but these undesirable mechanisms can be identified. According to our analysis there was no significant change in droplet size and/or PDI over the investigated period compared to initial values. The visual observation revealed no phase separation, creaming, or precipitation. The o/w baicalin containing nanoemulsion demonstrated high physical stability. The results of the long-term stability study are illustrated in Table 4. 

#### 3.5.6. Atomic Force Microscopy 

**Figure 5:**
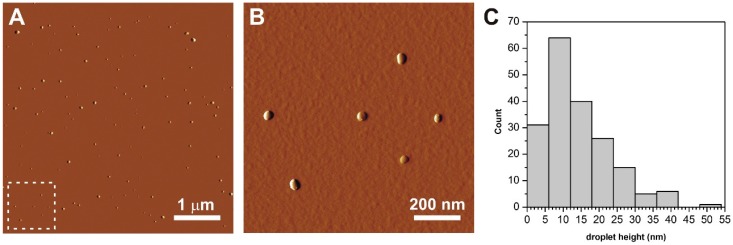
(**A**) Atomic force microscopy (AFM) amplitude-contrast images of surface adsorbed lyophilized oil droplets. (**B**) The area marked by dashed line is shown. (**C**) Height distribution of droplets (n = 122).

**Figure 6:**
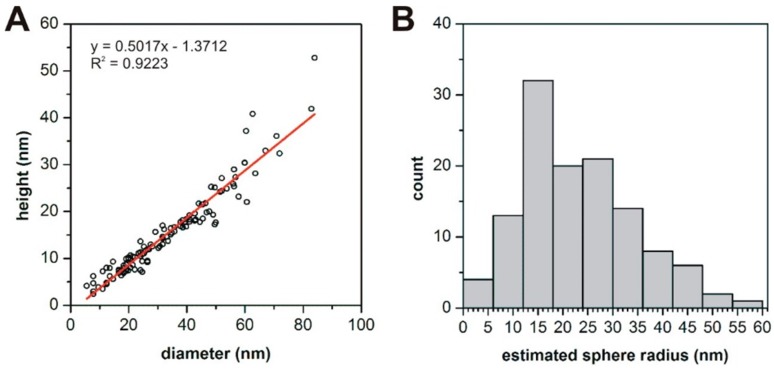
(**A**) Correlation between the height and diameter of surface-adsorbed oil droplets. (**B**) Histogram of estimated radius of oil droplets in the fluid phase. Estimation based on volume of surface-absorbed droplets (n = 122).

Lyophilized nanodroplets were dispersed homogeneously on a mica surface, showing no signs of aggregation (Figure 5A). They appeared as circular objects with smooth surfaces and a rounded topography (Figure 5B), which corresponds well to what one would expect from a surface adsorbed droplet. Height of particles followed a slightly right skewed distribution (Figure 5C). Lack of outstandingly large particle sizes indicate that droplets did not coalesce during sample preparation. Mean height of droplets (±SD) was 15.4 ± 9.0 nm, which was lower than the diameter (52.87 ± 0.5322 nm) obtained from DLS data. This may stem from two factors: (1) Flattening of emulsion droplets on the mica surface, or (2) the fact that DLS yields the hydrodynamic size of the droplets, which is bigger than the real particle diameter. Since the diameter of surface adsorbed droplets is about two times larger than their height (Figure 6A), at first sight one would think there was a considerable flattening indeed. However, a tip convolution artefact on its own can result in this extent of artefactual apparent widening of the vesicle diameter, with the tip radius being approximately 9 nm. 

To estimate the diameter of the original fluid-phase emulsion droplets, volume of surface-adsorbed droplets was determined. From this, the real diameter of fluid phase droplets could be calculated, assuming that they have spherical shape and there is no volume change upon surface-adsorption. Estimated fluid phase droplet radius showed an apparent normal distribution with 23.6 ± 12.0 nm as mean ± SD (Figure 6B). This value falls somewhat closer to the DLS size. The differences between them might be attributed to the hydrodynamic overestimation of particle size by the DLS method.

### 3.6. In Vitro Dissolution Study and Nanoemulsifying Ability Test of BSNEDDS

**Figure 7:**
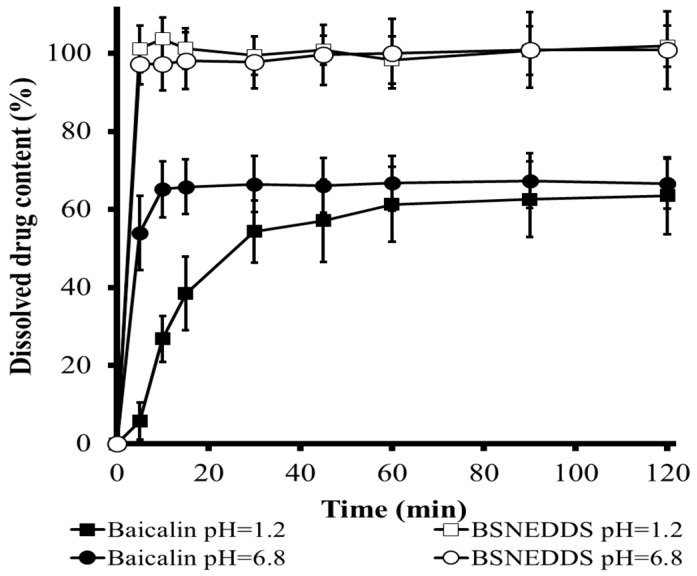
Dissolution profiles comparison of baicalin powder (filled symbol) and baicalin in optimized liquid SNEDDS (empty symbol) formulations at pH = 1.2 (square) and pH = 6.8 (circle). (Mean ± SD; n = 3).

In vitro dissolution profiles of BSNEDDS pre-concentrate and baicalin in pH = 1.2 and pH = 6.8 dissolution media are depicted in Figure 7. It was observed that baicalin loaded nanodroplets were rapidly dispersed in the media (within 5 min) irrespective of pH of the dissolution media, and 100% of baicalin was dissolved in nanodroplets. In contrast, the pure drug showed much lower dissolution rates for the initial period of 5 min at both pH values. It was proved that the dissolution rate of baicalin is significantly lower in the case of pH 1.2 correlated to pH 6.8, which can be explained by its pH-dependent solubility.

The liquid formula showed spontaneous nanoemulsification and there was no sign of phase separation or phase inversion of nanoemulsion after storage for 24 h. The nanoemulsion formation was successful because of the low PDI values (PDI_pH=1.2_: 0.321 ± 0.002, PDI_pH=6.8_: 0.347 ± 0.003) in both dissolution medias, indicating narrow droplet size distribution. The droplet size was also detected and showed adequate results: Z-avg_pH=1.2_: 84.93 ± 0.3235 nm, Z-avg_pH=6.8_: 86.67 ± 0.2956 nm. On the grounds of in vitro dissolution studies and reconstitution analysis, we can declare that a remarkable dissolution rate and solubilization improvement was achieved, and total amount of incorporated baicalin was dispersed in homogeneous size distribution nanoemulsified droplets.

## 4. Conclusions

Dissolution kinetics of baicalin was improved in a self-nanoemulsifying system taking different types of oils, surfactants, co-surfactants, and ideal ratio of components into consideration, based on response surface methodology, using a central composite experimental design. The best composition contains Peceol^®^ (14.29%, *w*/*w*), Kolliphor^®^ EL (57.14%, *w*/*w*), and Transcutol^®^ P (28.57%, *w*/*w*). Droplet size was measured by DLS method and confirmed by AFM, together with morphological characterization. A new sample preparation method gave the opportunity to analyze the lyophilized samples without any signs of aggregation of droplets. Droplet size after dispergation of BSNEDDS pre-concentrate was highly desirable, with 86.75 ± 0.3553 nm; the Zeta-potential was −24.3 ± 1.44 mV, which is also acceptable. The results proved that the low aqueous solubility and dissolution rate of baicalin can be significantly improved by the optimized BSNEDDS formula. The pre-concentrate showed very rapid emulsification (within seconds) and nanosized droplets were generated. The future research direction is to convert the optimized liquid BSNEDDS pre-concentrate to various solid carriers and preparing of self-nanoemulsifying matrix pellets using an extrusion-spheronization method.

## Figures and Tables

**Table 1:** Independent variables and their levels (**A**) and dependent variables with goals (**B**) used in face-centered experimental design.

**(A)**				
**Independent Variables**	**Symbol**	**Levels**		
		**−1**	**0**	**+1**
Oil:S_mix_ ratio	X_1_	1:8	1:6	1:4
Emulgent:co-emulgent ratio	X_2_	1:1	2:1	3:1
**(B)**				
**Dependent Variables**	**Symbol**	**Goal**		
Z-avg (nm)	Y_1_	Y_1_ < 200 nm		
Transmittance (%)	Y_2_	Y_2_ > 90%		
Zeta-potential (mV)	Y_3_	Y_3_ > ±20 mV		
Polydispersity index (PDI)	Y_4_	Y_4_ < 0.400		

**Table 2:** Summary of results of regression analysis for responses Z-avg, Transmittance, Zeta-potential, and PDI with R^2^, Adj-R^2^, and Pred-R^2^ tests (statistical significance indicated by * (*p* < 0.05)).

Source	Z-avg (nm)	Transmittance (%)	Zeta-Potential (mV)	PDI
***b*_0_**	+97.24	+96.74	−27.61	+0.46
***b*_1_**	+67.77 *	−8.33 *	+1.05 *	+0.17 *
***b*_2_**	−21.00 *	+1.63 *	−4.87 *	+0.030 *
***b*_12_**	−14.30 *	+2.45 *	−0.43	+0.045 *
***b*_11_**	−14.91 *	−5.58	+6.54 *	−0.045
***b*_22_**	+13.99	+0.72	−0.21	+0.035
**R^2^**	0.9885	0.9793	0.9728	0.9730
**Adj-R^2^**	0.9803	0.9645	0.9533	0.9537
**Pred-R^2^**	0.8887	0.8222	0.7732	0.9047

**Table 3:** Effect of pH and drug loading on Z-avg and PDI after dispergation of unloaded and loaded Self-nanoemulsifying formulations (Mean ± SD; n = 3).

**Z-avg (nm) ± SD**		
pH	*SNEDDS*	*BSNEDDS*
1.2	50.41 ± 0.321	84.93 ± 0.3235
6.8	52.84 ± 0.632	86.67 ± 0.2956
**PDI ± SD**		
pH	*SNEDDS*	*BSNEDDS*
1.2	0.231 ± 0.01	0.321 ± 0.002
6.8	0.217 ± 0.01	0.347 ± 0.003

**Table 4:** Results of long-term stability analysis of liquid baicalin loaded liquid self-nanoemulsifying drug delivery systems (BSNEDDS) formula. (Mean ± SD; n = 3).

Storage Intervals (days)	Z-avg (nm)	PDI
0	81.24 ± 0.17	0.406 ± 0.007
1	80.14 ± 0.08	0.405 ± 0.006
5	79.23 ± 0.14	0.379 ± 0.002
7	82.37 ± 0.10	0.400 ± 0.002
21	79.53 ± 0.05	0.443 ± 0.003
28	80.86 ± 0.28	0.389 ± 0.003
